# Identification of a heterogeneous and dynamic ciliome during embryonic development and cell differentiation

**DOI:** 10.1242/dev.201237

**Published:** 2023-04-27

**Authors:** Kelsey H. Elliott, Sai K. Balchand, Christian Louis Bonatto Paese, Ching-Fang Chang, Yanfen Yang, Kari M. Brown, Daniel T. Rasicci, Hao He, Konrad Thorner, Praneet Chaturvedi, Stephen A. Murray, Jing Chen, Aleksey Porollo, Kevin A. Peterson, Samantha A. Brugmann

**Affiliations:** ^1^ Division of Developmental Biology, Department of Pediatrics, Cincinnati Children's Hospital Medical, Cincinnati, OH 45229, USA; ^2^ University of Cincinnati, College of Medicine, Department of Pediatrics, Cincinnati, OH 45229, USA; ^3^ The Jackson Laboratory, Bar Harbor, ME 04609, USA; ^4^ Division of Biomedical Informatics, Cincinnati Children's Hospital Medical, Cincinnati, OH 45229, USA; ^5^ Center for Autoimmune Genomics and Etiology, Cincinnati Children's Hospital Medical, Cincinnati, OH 45229, USA; ^6^ Division of Plastic Surgery, Department of Surgery, Cincinnati Children's Hospital Medical Center, Cincinnati, OH 45229, USA

**Keywords:** Primary cilia, Tissue heterogeneity, Craniofacial, Skeletogenesis, Ciliopathy

## Abstract

Primary cilia are nearly ubiquitous organelles that transduce molecular and mechanical signals. Although the basic structure of the cilium and the cadre of genes that contribute to ciliary formation and function (the ciliome) are believed to be evolutionarily conserved, the presentation of ciliopathies with narrow, tissue-specific phenotypes and distinct molecular readouts suggests that an unappreciated heterogeneity exists within this organelle. Here, we provide a searchable transcriptomic resource for a curated primary ciliome, detailing various subgroups of differentially expressed genes within the ciliome that display tissue and temporal specificity. Genes within the differentially expressed ciliome exhibited a lower level of functional constraint across species, suggesting organism and cell-specific function adaptation. The biological relevance of ciliary heterogeneity was functionally validated by using Cas9 gene-editing to disrupt ciliary genes that displayed dynamic gene expression profiles during osteogenic differentiation of multipotent neural crest cells. Collectively, this novel primary cilia-focused resource will allow researchers to explore longstanding questions related to how tissue and cell-type specific functions and ciliary heterogeneity may contribute to the range of phenotypes associated with ciliopathies.

## Teaser

**Summary:** A searchable transcriptomic resource for a manually curated primary ciliome, detailing various subgroups of differentially expressed genes within the ciliome that display tissue and temporal specificity.

## INTRODUCTION

Cilia are microtubule-based cellular organelles common to almost all eukaryotic cells across the animal kingdom (Goetz and Anderson, 2010). Cilia perform vital functions during several biological processes, including locomotion, left-right patterning, vision, olfaction, airway clearance and reproduction (Choksi et al., 2014). For all cilia, there is a highly conserved and recognized basic structure that consists of a ciliary membrane ensheathing a microtubule core (axoneme) and a specialized centriolar structure that anchors the cilium to the cell surface (the basal body). Together, these ciliary compartments are necessary to convey both molecular- and mechano-signal transduction capabilities upon the organelle (Pazour et al., 2002, 2000).

Ciliopathies are a class of diseases that arise when the structure or function of the cilium is compromised. Although mutations that prevent ciliogenesis often result in early embryonic lethal phenotypes (Eggenschwiler and Anderson, 2007), mutations that more moderately impact ciliary function are viable. Frequently, mutations such as these result in impaired or excessive signaling of molecular pathways required for development (Goetz and Anderson, 2010). Thus, a complete understanding of the cadre of genes and proteins that are necessary and sufficient for ciliary functions, referred to herein collectively as the ciliome, holds great biomedical relevance. To date, several studies have been performed to comprehensively demarcate the ciliome. Syscilia Gold standard (van Dam et al., 2013), CiliaCarta (van Dam et al., 2019) and Cil*db* (Arnaiz et al., 2014, 2009; Reiter and Leroux, 2017) represent three independent studies dedicated to defining the ciliome. In addition to these undertakings, ciliogenesis modulator screens have been carried out to identify drivers and repressors of ciliogenesis (Kim et al., 2010; Wheway et al., 2015). Although these studies have ushered in a new era in studying the cilium, they have yet to be integrated to increase our understanding of the wide spectrum of phenotypes associated with ciliopathic conditions.

Several observations and experimental findings suggest a degree of heterogeneity beyond the structural aspect of the cilium that has yet to be appreciated. First, ciliopathies present with a wide variety of tissue-specific phenotypes. For example, Joubert syndrome (JS) and Bardet Biedl Syndrome (BBS) present with variable CNS and neuropsychiatric phenotypes, while Oral-Facial-Digital syndrome, Short Rib Polydactyly (SRP) and Meckle-Gruber syndrome (MKS) all present with skeletal defects (Cortés et al., 2016; Gorlin et al., 1990). This degree of phenotypic variation suggests that ciliopathic mutations have tissue-specific impacts across vertebrate species. Second, the loss of functional cilia does not always produce a uniform molecular readout across tissues. For example, several intraflagellar transport (IFT) mutants present with a loss-of-Hedgehog phenotype in the developing neural tube and gain-of-Hedgehog phenotype during limb development (Huangfu and Anderson, 2005; May et al., 2005). Within the craniofacial complex, loss of the ciliary gene kinesin family member 3a (*Kif3a*) results in a gain-of-Hedgehog or loss-of-Hedgehog signaling in the frontonasal and mandibular prominences, respectively (Chang et al., 2016; Millington et al., 2017). Taken together, these isolated experimental studies support the hypothesis that there may be a previously unappreciated amount of temporal, spatial and biologically relevant heterogeneity within the ciliome. A comprehensive analysis of ciliome heterogeneity between tissues commonly affected in ciliopathies has yet to be performed.

To definitively determine the extent of heterogeneity within the ciliome, we compared the ciliome of six distinct embryonic domains, including those that are commonly impacted in ciliopathies (neural, limb and craniofacial). Using unbiased transcriptional profiling and established ciliary databases (Arnaiz et al., 2014, 2009; Kim et al., 2010; Reiter and Leroux, 2017; van Dam et al., 2013, 2019; Wheway et al., 2015), our data comprehensively reveal that ∼30% of the ciliome is differentially expressed across analyzed tissues in the developing embryo and genes that are a part of the differentially expressed ciliome are under less stringent functional and evolutionary constraint. Furthermore, we profiled expression of the ciliome during differentiation of multipotent cranial neural crest cells and observed upregulation of numerous ciliary genes correlating with osteogenic cell fate decisions, suggesting that changes in the ciliome contribute to distinct functions of cell types in vertebrate species.

## RESULTS

### A unique subclass of differentially expressed ciliary genes suggests ciliary heterogeneity

**Fig. 1.**
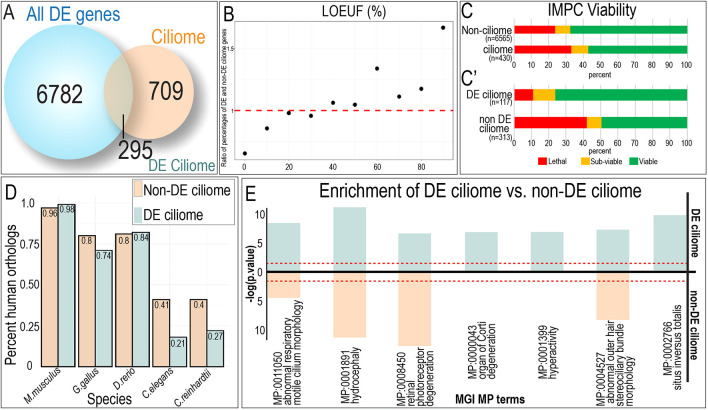
**Transcriptomes of developing craniofacial tissues reveal differential expression of ciliary genes.** (A) Venn diagram depicting the overlap between the combined number of differentially expressed genes between neural, facial and limb samples (blue), and curated ciliome (orange). (B) Ratio of loss of function observed over expected upper bound fraction (LOEUF) percentages of DE and non-DE ciliome genes. (C) Summary of primary viability screening data from the International Mouse Phenotyping Consortium (IMPC) for non-ciliome, ciliome, DE ciliome and non-DE ciliome gene sets. Lethal, subviable and viable bins are defined from intercrosses between heterozygous animals and then genotyping offspring (*n*>28) to determine the number of homozygous animals present at weaning. (D) Graphical analysis of the percentage sequence similarity between genes within the non-DE ciliome and the DE ciliome between various species. (E) Enrichment analysis based on MGI MP terms and comparison between DE and non-DE ciliome. FDR≤1E-04.

To unbiasedly determine the extent of heterogeneity among cilia across selected embryonic tissues, we used datasets that comprehensively profiled the ciliome and screened for modulators of ciliogenesis (Arnaiz et al., 2014, 2009; Kim et al., 2010; Reiter and Leroux, 2017; van Dam et al., 2013, 2019; Wheway et al., 2015). Curation, of these datasets generated a comprehensive list of 1004 unique and previously verified protein-coding ciliary genes collectively referred to here as the ciliome (Table S1; https://research.cchmc.org/Ciliome_Gene_Expression/). Despite some overlap, our curated ciliome was distinct from the recently published transcriptome of motile cilia (Patir et al., 2020) and primary cilia proteomes from mouse IMCD3 cells (Ishikawa et al., 2012; May et al., 2021; Mehta et al., 2022; Mick et al., 2015) (Table S1). We examined tissues from organ systems severely affected in ciliopathies, including the developing face, brain and limbs. Bulk RNA-seq analysis was performed on three dissected facial tissue samples from embryonic day 11.5 (E11.5) mouse embryos: the frontonasal prominence (FNP), the mandibular prominence (MNP) and the maxillary prominence (MXP). For comparison, E11.5 limb, dorsal brain (dB) and ventral brain (vB) samples from the developing forebrain and midbrain regions were also collected. Tissue samples were pooled for each biological replicate (*n*≥5) to reduce gene expression variations between individual embryos. RNA-seq sample distribution was visualized in a t-distributed stochastic neighbor embedded (t-SNE) plot. Individual samples for each tissue clustered closely, demonstrating low variation and high reproducibility. As expected, facial tissues (MXP, MNP and FNP) were more closely related to each other than to neural tissues (dB and vB) (Fig. S1A). Comparison between any two of the six tissue samples collected revealed a total of 7077 differentially expressed (DE) genes with a fold change of at least 2.0 and a 5% false discovery rate (FDR) (Fig. 1A).

To determine differential expression of the ciliome between embryonic tissues, 7077 total differentially expressed genes were intersected with 1004 genes of the ciliome. The comparison revealed that 295/1004 (∼29%) of previously identified ciliary genes were differentially expressed between any two tissues (Fig. 1A; Table S1). These 295 differentially expressed genes (referred to as the DE ciliome hereafter) represented all ciliary compartments, including the basal body, transition zone, axoneme and ciliary membrane (Fig. S1B). Phenotype/disease enrichment of the DE ciliome identified several phenotypes such as ciliopathies, situs inversus and Kartagener syndrome/primary ciliary dyskinesia associated with these genes, further confirming their ciliary functionality (Fig. S1C).

Although *in vivo* transcriptomic analysis supported the concept of ciliary heterogeneity within the embryo, *in vitro* data in cell types that represented organ systems frequently affected in ciliopathies (NIH-3T3-fibroblasts, NE4C-neuroectodermal and O9-1-neural crest) (Ishii et al., 2012; Schlett et al., 1997; Todaro and Green, 1963) revealed additional variation in ciliary length, number and rate of ciliary extension. Immunostaining for the axonemal marker Arl13b (Caspary et al., 2007) demonstrated that neural crest cells possessed the longest cilia and highest rate of ciliation, followed by fibroblasts and neuroectodermal cells, respectively (Fig. S1D,D′). Rates of ciliary extension, as induced by cytochalasin D treatment, also varied significantly across cell populations, with fibroblasts exhibiting the most rapid rates of extension (Fig. S1D,D′).

To determine whether embryonic ciliary heterogeneity was biologically relevant, we assayed viability associated with mutations across the ciliome in both human and mouse via the Genome aggregation database (gnomAD) (Karczewski et al., 2020) and the International Mouse Phenotyping Consortium (IMPC) (Cacheiro et al., 2020; Dickinson et al., 2016), respectively. 928 of the 1004 ciliome genes had gnomAD constraint scores. The loss-of-function observed/expected upper bound fraction (LOEUF) scores of ciliome genes (mean=0.802) were significantly lower compared with the rest of the genome (mean=0.960; *t*-test *P*-value=6.93^−24^), suggesting ciliome genes were under selection. Genes of the DE ciliome (*n*=272, mean=0.861) were compared with genes of the non-DE ciliome (*n*=656, mean=0.776), and had significantly higher LOEUF scores (*t*-test *P*-value=0.0096), suggesting DE ciliome genes were under less selection than the non-DE ciliome genes.

To further examine this difference in mean LOEUF, the ratio of percentages for genes of the DE ciliome over genes of the non-DE ciliome was calculated for each decile (Fig. 1B; Table S2). This analysis showed a clear trend where the ratio of DE compared with non-DE ciliome was lower at lower LOEUF (higher constraint) and increased at higher LOEUF (less constraint), providing further evidence that genes in the non-DE ciliome are under a greater level of selective pressure than genes in the DE ciliome. Furthermore, GO-term analysis of the non-DE ciliome revealed that the most significant associations were with structural components of the ciliome (e.g. centrosome, axoneme, ciliary tip and ciliary transition zone; Table S3). Together, these data support the hypothesis that the non-DE ciliome is more highly constrained because it represents a cohort of genes essential for ciliogenesis and ciliary function, while the DE ciliome is less constrained because it contributes to tissue-specific functions.

In addition to evidence of functional constraint based upon human sequence data, large-scale primary viability screening in knockout mice provides another source of information to assess gene essentiality. First, to validate the importance of the ciliome in total, we compared the viability of murine embryos with mutations in genes within and outside the ciliome, as determined by the IMPC. For genes analyzed by the IMPC, mutations in ciliary genes were less tolerated relative to the rest of the genome, with over 40% classified as essential (lethal and subviable) (Fig. 1C). Second, the percentage of lethal genes associated with mutations in the DE ciliome was lower than non-DE ciliome, with only ∼10% of DE ciliome mutations resulting in lethality when compared with >40% within the non-DE ciliome (Fig. 1C′). These findings agree with the LOEUF analysis supporting lower levels of constraint for DE genes. Furthermore, statistical analysis revealed that genes within the non-DE ciliome were enriched for the mammalian phenotype (MP) term ‘preweaning lethality, complete penetrance’ when compared with the full ciliome (Fig. S1E). Together, these data identified a defined subset of the ciliome that lacked ubiquitous expression and was dispensable for essential functions related to ciliogenesis. Interestingly, when analyzing orthologs from the PANTHER database (Thomas et al., 2022), genes of the DE ciliome had fewer orthologs from non-vertebrates (*C. elegans* and *C. reinhardtii*, Fig. 1D)*.* As genes conserved across phyla are more likely to carry out essential core biological functions, and clade-specific genes are more likely to perform organism- or cell type-specific tasks (Mata and Bahler, 2003), these data collectively suggested that the DE ciliome represents a distinct class of genes with cell- or tissue-specific function. Enrichment analysis based on MGI MP terms revealed several specific phenotypes significantly enriched within the DE ciliome relative to the non-DE ciliome (Fig. 1E). Genes associated with hyperactivity, organ of Corti degeneration, impaired mucociliary clearance and situs inversus totalis were significantly enriched within the DE ciliome versus the non-DE ciliome, further supporting the tissue-specific biological relevance of the DE ciliome. Thus, using several measures of analysis to characterize associated phenotype, molecular function and cellular compartment, the DE-ciliome is distinct from the non-DE ciliome (Tables S1 and S3).

### The differentially expressed ciliome contributes to tissue specificity

**Fig. 2.**
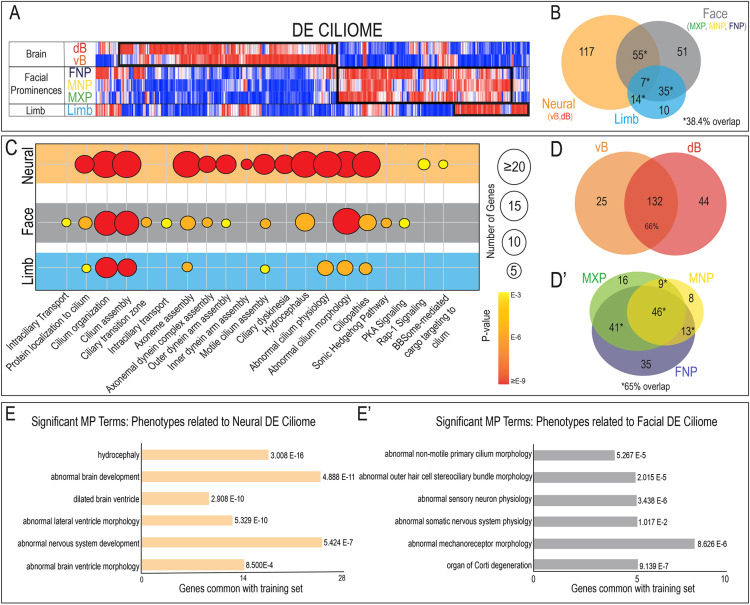
**Ciliary heterogeneity correlates with tissue of origin.** (A) Heatmap showing hierarchical clustering of DE ciliary genes separated according to neural, facial and limb tissues. (B) Venn diagram depicting the genes upregulated in neural, facial, limb and possible combinations of tissues. (C) Summary of GO terms enriched in ciliary genes robustly expressed in neural, facial or limb tissues. Bubble size indicates the number of ciliary genes per annotation, color reflects range of *P*-values. (D) Venn diagram depicting the overlap between genes of the DE ciliome upregulated within ventral (vB) and dorsal (dB) neural tissue. (D′) Venn diagram depicting the overlap between genes of the DE ciliome upregulated within the facial prominences: frontonasal prominence (FNP), mandibular prominence (MNP) and maxillary prominence (MXP). (E,E′) Representative tissue-specific mammalian phenotype (MP) terms enriched in the neural (E) and facial (E′) DE ciliome gene sets (FDR<0.05).

Initial analysis of RNA-seq data revealed tissue replicates exhibited a more similar transcriptomic profile than unrelated tissue samples (Fig. S1A). Concordantly, examination of the DE ciliome revealed that the cohort of differentially expressed genes was similar between closely related tissues (Fig. 2A). Neural tissues (dB and vB) had a distinct pattern of ciliary gene expression when compared with facial prominences (FNP, MXP and MNP) and limb tissue. Of the 295 genes of the DE ciliome that were differentially expressed across dB, vB, FNP, MXP, MNP and limb, 289 were differentially expressed when compressing the six isolated embryonic structures into three general tissue categories: neural (dB and vB), facial (FNP, MXP and MNP) and limb. Pair-wise comparison between each tissue identified that 3.4% (10/289) of DE ciliary genes were exclusively upregulated in the limb, whereas 12% (35/289) or 4.8% (14/289) of DE ciliary genes were upregulated in both limb and facial tissues or limb and neural tissues, respectively (Fig. 2B; Table S1). In the face, 17.6% (51/289) of DE ciliary genes were solely upregulated in facial tissues (FNP, MXP and MNP), whereas 12% (35/289) of DE ciliary genes were upregulated in both facial tissues and limb tissues, and 19% were upregulated in both facial and neural tissues (55/289; Fig. 2B; Table S1). Finally, 40% (117/289) of DE ciliary genes were upregulated exclusively in neural tissues (Fig. 2B; Table S1). Thus, there was a higher percentage of DE ciliome genes expressed exclusively in one tissue type (∼62%) than DE ciliome genes with overlapping expression across at least two tissues (∼38%; Fig. 2B).

ToppGene Suite (Chen et al., 2009a,b, 2007) analysis suggested that the DE ciliome contributed to tissue-specific cellular processes among neural, facial and limb samples. Although terms like ‘cilium organization’ and cilium ‘assembly’ were associated with all three tissues, terms related to formation and function of motile cilia (‘inner dynein arm assembly’, ‘outer dynein arm assembly’ and ‘motile cilia assembly’) were enriched in neural tissues relative to facial and limb tissues (Boon et al., 2014; Brody et al., 2000; Moore et al., 2013; Patir et al., 2020; Zou et al., 2020) (Fig. 2C; Fig. S2A). Given the fact that the neural tissues were the only tissues to contain motile cilia, these results were expected and served as proof-of-principle and quality control for our approach. ToppGene Suite analysis of the neural DE ciliome generated GO terms related to molecular function and highlighted a significant enrichment in genes that contribute to tubulin binding and glutamylation, including tubulin tyrosine ligase-like (Ttll) genes (Fig. S2B). Furthermore, there was a higher percentage of overlapping expression of neural DE ciliary genes within neural tissues (ventral versus dorsal) than related tissues, with 66% (132/201) of genes upregulated in both ventral and dorsal neural tissue (Fig. 2D; Fig. S2A). Thus, our unbiased bioinformatic analysis supported the hypothesis that the DE ciliome contributed to the formation of tissue-specific cilia.

To validate this hypothesis, we next examined the extent to which the DE ciliome varies between the different facial prominences (FNP, MXP and MNP). This comparison identified the FNP and MXP as the two prominences containing the greatest number of unique genes (FNP, *n*=35; MXP, *n*=16); however, more than half of all the facial DE ciliome genes (109/168) were either shared by all three prominences or between any two (Fig. 2D′; Table S1). ToppGene Suite analysis of the facial DE ciliome generated GO terms related to molecular function, indicating a significant enrichment in genes that contribute to cytoskeletal and actin filament binding (Fig. S2C). In summary, there was a higher percentage of overlapping expression of DE ciliary genes within regions isolated from the same tissue (face and neural) versus between tissues.

To determine whether mutations within the DE ciliome were biologically relevant, MP term enrichment was performed on tissue-specific DE ciliome gene sets. For the neural DE ciliome, this analysis identified phenotypes including ‘hydrocephaly’, ‘abnormal brain development’, ‘abnormal ventricles’ and ‘abnormal nervous system development’ (Fig. 2E). Conversely, when this analysis was repeated for the facial DE ciliome, a distinctly different set of terms were returned. Notably, MP terms related to inner ear development (‘abnormal outer hair cell stereociliary bundle morphology’ and ‘organ of Corti degeneration’) and neural crest derived neurons (‘abnormal sensory neuron physiology’) were enriched in the facial populations. Furthermore, enrichment of the ‘abnormal mechanoreceptor morphology’ was also distinct to the facial DE ciliome (Fig. 2E′). Although the low number of limb-specific genes prevented enrichment analysis, data from neural and facial samples supported the hypothesis that the DE ciliome was not only distinct between tissues, but also participated in conveying tissue-specific identity and phenotypes.

**Fig. 3.**
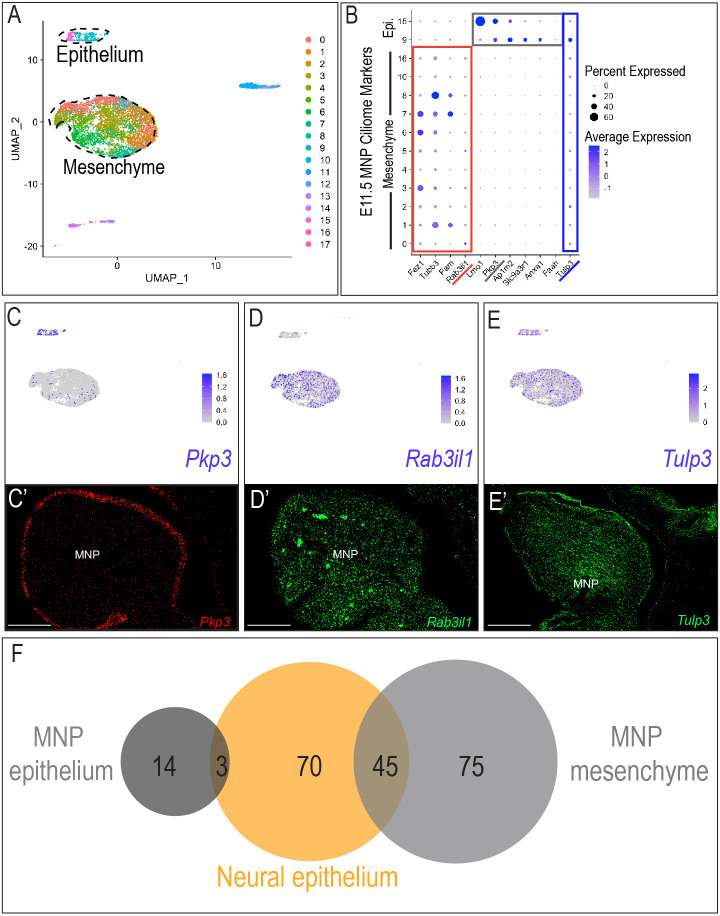
**Single cell analysis confirms ciliary heterogeneity.** (A) UMAP plot of cell clusters present in E11.5 MNP. (B) Dot plot highlighting ciliary genes expressed in mesenchymal (red), epithelial (gray) or epithelial and mesenchymal (blue) clusters. (C-E′) Feature plots and RNAScope in the E11.5 MNP for *Pkp3*, *Rab3il1* and *Tulp3*. (F) UpSetR visualization of the intersection between MNP epithelium, neural epithelium and MNP mesenchyme gene sets. Scale bars: 100 μm.

Despite the fact that our supposition that the ciliome was distinct between tissues had previously been supported (Bangs and Anderson, 2017), we next tested an alternative hypothesis – that the ciliome was cell type specific (e.g. epithelium versus mesenchyme) – as neural samples were epithelial, and facial and limb samples were predominantly mesenchymal in nature. To address this possibility, we examined Uniform Manifold Approximation and Projection (UMAP) plots generated from scRNA-Seq experiments (Elliott et al., 2020) of the mandibular prominence (MNP). As the MNP is mostly composed of cranial neural crest (CNCC)-derived mesenchyme, surrounded by ectodermally derived epithelium, two distinct groups of cell clusters were clearly identified (Fig. 3A). The large central group of multiple clusters represented mesenchymal cells, mostly neural crest in origin, as evident by the robust expression of neural crest marker *Snai1*, whereas a smaller cluster representing epithelial cells, as evident by robust expression of the marker *Epcam* (Fig. S3A,A′). We examined expression of the DE ciliome across mesenchymal and epithelial clusters with the expectation that if the DE ciliome was indeed related to cell type, rather than tissue-specific function, there would be a greater overlap between genes expressed in the MNP epithelium and neural samples than between the MNP epithelium and MNP mesenchyme. Distinct transcriptomic profiles were observed between the MNP epithelium and mesenchyme. Several genes were almost exclusively expressed in the mesenchymal clusters, including *Fez1*, *Tubb3*, *Pam* and *Rab3il1* (Fig. 3B; Table S1), while others including *Lmo1*, *Pkp3*, *Ap1m2*, *Slc9a3r1*, *Anxa1* and *Faah* were more robustly expressed in epithelial clusters (Fig. 3B; Table S1). There was also a subset of genes that was expressed in both mesenchymal and epithelial clusters (e.g. *Tulp3*) (Fig. 3B; Table S1). Epithelial versus mesenchymal expression was validated for three genes that represented epithelial-specific, mesenchymal-specific or epithelial- and mesenchymal-specific expressed genes. For example, expression of plakophilin3 (*Pkp3*), a cytoskeleton interacting protein-coding ciliary gene and a negative regulator of ciliogenesis (Kim et al., 2010; Munoz et al., 2012) was robustly detected in epithelial defined cell clusters (Fig. 3C) and its expression was confined to the epithelium of the developing MNP (Fig. 3C′). Rab3a interacting like protein 1 (*Rab3il1*), which encodes a Rab8-binding GEF (Westlake et al., 2011) was barely detectable in the epithelial cluster, yet was expressed throughout the mesenchymal cluster (Fig. 3D) and its expression was restricted to the mesenchyme of the MNP (Fig. 3D′). Tubby like protein 3 (*Tulp3*), a gene encoding a tubby like family protein that interacts with the IFT-A complex and functions as a negative regulator of the Shh pathway (Mukhopadhyay et al., 2010; Norman et al., 2009), was expressed within both mesenchymal and epithelial clusters (Fig. 3E), and, as such, its expression was observed throughout both epithelial and mesenchymal tissues of the MNP (Fig. 3E′). Comparison of DE ciliome genes expressed in the MNP epithelium and MNP mesenchyme with DE ciliome genes expressed in the neuroepithelium revealed a higher percentage of overlap between MNP mesenchyme and neuroepithelium than MNP epithelium and neuroepithelium (Fig. 3F), suggesting that differences observed between neural and facial tissues were not due to the epithelial versus mesenchymal character of tissue samples.

Finally, to confirm that observed transcriptional expression was maintained on a translational level, E11.5 wild-type MNPs were dissected to isolate either surface ectoderm or mesenchyme (Li and Williams, 2013). Western blot analysis on pooled epithelial (surface/facial ectoderm) and mesenchymal (neural crest) samples confirmed tissue-specific expression of selected ciliary proteins (Fig. S3B). Pkp3 was enriched in epithelial lysates, whereas Rab3il1 was enriched in the mesenchymal lysates. Consistent with scRNA-seq and RNA-scope results, Tulp3 was enriched in both epithelial and mesenchymal fractions. Together, these experiments supported the conclusion that the DE ciliome contributed to tissue-specific identity at both a transcriptional and translational level.

### The ciliome is dynamic during cell differentiation

**Fig. 4.**
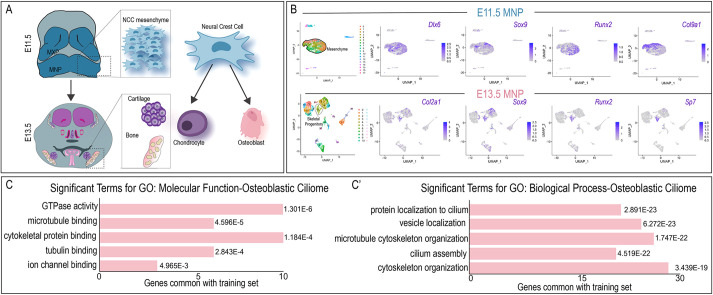
**The ciliome is dynamic during cell differentiation.** (A) Schematic of E11.5 and E13.5 heads, noting neural crest cell mesenchyme at E11.5 and neural crest skeletal derivatives at E13.5. (B) Feature plots of marker genes for skeletal progenitors in the E11.5 and E13.5 mandibular prominences (MNPs). (C,C′) GO-term enrichment analysis for genes upregulated in the osteogenic ciliome (FDR<0.05).

**Fig. 5.**
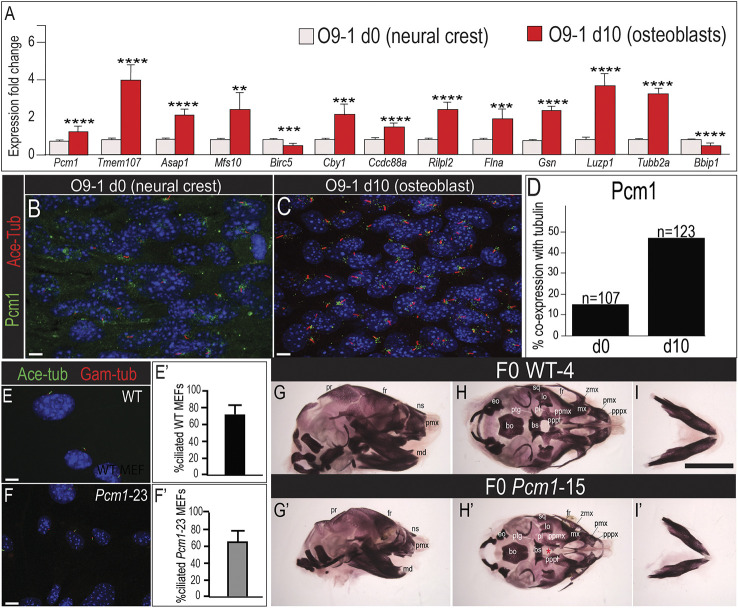
**Disruptions to the osteogenic ciliome result in skeletal phenotypes.** (A) qPCR analysis for select genes of the osteogenic ciliome from d0 O9-1 NCCs and d10 O9-1-derived osteoblasts. (B,C) Co-immunostaining for acetylated-tubulin and Pcm1 in d0 O9-1 NCCs and d10 O9-1-derived osteoblasts. (D) Quantification of cells co-expressing acetylated-tubulin and Pcm1. (E,F) Immunostaining for acetylated-tubulin and γ-tubulin on MEFs derived from unedited wild-type and mutant F0 *Pcm1*-23 embryos. (E′,F′) Quantification of the percentage of ciliated cells in wild-type and *Pcm1*-23 MEFs. (G-I′) Lateral (G,G′), ventral (H,H′) and mandibular (I,I′) views of Alizarin Red stained skulls and mandibles from E17.5 wild-type and F0 *Pcm1*-15. Two-tailed unpaired *t*-tests were performed, *P≤0.05, **P≤0.01, ***P≤0.001 and ****P≤0.0001. Data are mean±s.e.m. Scale bars: 10 µm in B,C,E,F; in I, 3 mm for G-I′. Red asterisk in H′ indicates cleft palate. pr, parietal; ns, nasal; md, mandible; eo, exoccipital; bo, basioccipital; bs, basisphenoid; ptg, pterygoid; pl, palatine; lo, lamina obturans; sq, sqamosal; fr, frontal; zmx, zygomatic process of maxilla; mx, maxillary; ppmx, palatal process of maxilla; pppl, palatal process of palatine; pppx, palatal process of premaxilla; pmx, premaxilla.

Transcriptomics data supported the hypothesis that although a majority of the ciliome was ubiquitously expressed, a subgroup was expressed in a tissue-specific manner across the embryo. Based on these data, we further challenged the concept of ciliary heterogeneity temporally by assaying variation of the full ciliome during cell differentiation focusing on MNP development. Cranial neural crest cells (NCCs) are a multipotent, mesenchymal cell population capable of differentiating into a multitude of derivatives, including osteoblasts (Ramaesh and Bard, 2003; Shibata et al., 2006) (Fig. 4A). In contrast to E11.5 MNP NCCs (marked by the expression of *Dlx6*, *Sox9*, *Runx2* and *Col9a1*), which organized into a large unseparated population, NCC-derived skeletal progenitors within the E13.5 MNP occupied a distinct cluster (Fig. 4B). The increased separation of clusters in NCCs at E13.5 suggested that differentiation programs of the multipotent NCC population had commenced. Cluster 5 within the E13.5 MNP was identified as NCC-derived osteoprogenitors based on the expression of runt-related transcription factor 2 (*Runx2*) and osterix (*Sp7*)*,* which are master regulators of osteogenic lineage (Banerjee et al., 1997; Elliott et al., 2020; Nakashima et al., 2002; Prince et al., 2001) (Fig. 4B). To assay for potential changes in the ciliome during NCC differentiation, we compared expression of our ciliome (Table S1) between E11.5 mesenchymal clusters and the E13.5 skeletal progenitor cluster (E13.5 cluster 5) (Fig. S4A). Several ciliary genes showed at least 20% change in the number of cells expressing them (either increased or decreased) between E11.5 and E13.5 samples (Fig. S4B; Table S1). Interestingly, of the ciliary genes that had significant expression changes during osteogenic differentiation, nine were previously identified in the DE ciliome and 36 were not. Ciliary genes exhibiting expression changes during osteoblastic differentiation were associated with skeletal ciliopathies (Meckel-Gruber syndrome, Meckel syndrome type 2, Meckel syndrome type 13 and orofaciodigital syndrome type 16), and other skeletal pathologies, including frontometaphyseal dysplasia (*Flna*), spondylometaphyseal dysplasia (*Trip11* and *Ift20*), osteofibrous dysplasia (*Tmem107* and *Tmem216*) and polydactyly (*Bbip1*, *Flna*, *Arl3*, *Tmem107*, *Cby1*, *Cep164*, *Lztfl1* and *Tmem216*) (data not shown). Furthermore, analysis of GO terms for molecular function and biological processes revealed an enrichment of genes associated with microtubule and cytoskeletal binding/organization, as well as vesicle and protein localization to the cilium (Fig. 4C,C′). To confirm changes in gene expression during osteogenesis, we selected genes within the osteoblastic ciliome and examined expression during *in vitro* differentiation of the O9-1 murine NCC line (Ishii et al., 2012) and/or human induced pluripotent stem cell (iPSC)-derived NCCs. Using previously established osteogenic protocols (Ishii et al., 2012), O9-1 and hiPSC-derived NCCs were differentiated into osteoprogenitors over a 10-, 12- or 21-day period. Alizarin Red and/or hydroxyapatite (HA) staining and increased expression of *Runx2,* osteocalcin (*Ocl*) and alkaline phosphatase (*Alp*) confirmed osteogenic differentiation (Fig. S4C-D″). qPCR analysis determined that expression of several genes was significantly changed in murine or human osteoblasts when compared with undifferentiated O9-1 or hiPSC-derived NCCs (Fig. 5A; Fig. S5A). To address expression changes at the translational level, immunostaining for pericentriolar material 1 (Pcm1), a component of centriolar satellites that is essential for the correct localization of several centrosomal proteins and for the anchoring of microtubules to the centrosome, was performed on undifferentiated O9-1 and O9-1-derived osteoblasts. Relative to undifferentiated O9-1 NCCs, Pcm1 expression was increased at the base of the axoneme in O9-1-derived osteoblasts (Fig. 5B-D). Interestingly, expression levels of other core ciliary proteins, not determined to be a part of the osteogenic ciliome (Cep135), did not display increased expression in O9-1 osteoblasts when compared with undifferentiated O9-1 NCCs (Fig. S5B-D).

Although expression of genes and proteins within the osteoblastic ciliome was validated through various assays, biological relevance of these changes remained unclear. To confirm biological relevance, CRISPR/Cas9-edited F0 embryos were generated for two genes that displayed a significant increase in expression during osteogenic differentiation: *Pcm1* and *Tmem107*. Despite a previous report about mice haploinsuffient for *Pcm1* (Zoubovsky et al., 2015), homozygous null mutants have not been extensively studied outside the standard IMPC phenotyping pipeline. Conversely, *Tmem107* mutants have been previously studied as both an ENU allele and a homozygous null (Cela et al., 2018; Christopher et al., 2012); thus, we used *Tmem107* as a proof-of-principle experiment to validate our CRISPR/Cas9 F0 knockout approach (Fig. S5E). Isolated MEFs from *Pcm1-* or *Tmem107*-edited F0 embryos were stained for cilia extension via co-expression of acetylated tubulin (axoneme) and γ-tubulin (basal body). *Pcm1*-edited MEFs displayed a comparable number of cells extending a cilium relative to wild-type MEFs (Fig. 5E-F′). Although *Tmem107-*edited MEFs displayed a reduced number of cells extending a cilium, ∼25% of cells still did (Fig. S5F-F′). In contrast, loss of core components of the ciliome, such as Cep135, resulted in fewer than 10% of cells extending a cilium (Fig. S5G,G′).

Further examination of *Pcm1*- and *Tmem107*-edited F0 embryos was performed later at E17.5 to assess their impact on skeletal development (Fig. 5G-I′; Fig. S5H-K). In the most highly Cas9-edited *Pcm1* F0 embryo, almost all neural crest-derived bones were hypoplastic (Fig. 5G-I′). Notably the palatal processes of the palatine were reduced in size to the extent that they failed to fuse across the midline (Fig. 5H′, red asterisk). The mandible was also reduced in size (Fig. 5I′). There was variability in the severity of the embryonic phenotype associated with *Pcm1* Cas9-edited embryos, consistent with findings from the IMPC phenotyping pipeline reporting *Pcm1* as a subviable line displaying variable penetrance in abnormal cranium morphology in homozygous animals (https://www.mousephenotype.org/data/genes/MGI:1277958). Notably, *Pcm1* homozygous mutants consistently display reduced body length compared with wild-type controls, further suggesting additional impact on skeletal development. For *Tmem107*, Cas9-edited F0 embryos phenocopied skeletal phenotypes previously reported in *Tmem107^−/−^* embryos (Cela et al., 2018), presenting with reduced ossification of frontal, parietal and interparietal bones, and hypoplastic nasal, maxillae and pre-maxillae bones. Notably, the premaxillae were fused across the facial midline (Fig. S5H-J; white asterisk) and the basisphenoid and palatal processes were cleft along the midline axis (Fig. S5I; red asterisks). The mandible was also reduced in size (Fig. S5J) and facial clefting was observed (Fig. S5K). Together, these data suggest that genes of the osteogenic ciliome are more dispensable for ciliogenesis than core components of the ciliome, yet remain required for the ciliary function that is necessary for proper skeletal development.

## DISCUSSION

Based on numerous studies reporting ciliopathies with tissue-specific phenotypes and unique molecular readouts, we sought to explore the extent of *in vivo* ciliome heterogeneity in both spatial and temporal contexts during embryonic development and to determine whether differences in the ciliome correlated with biological function. Using unbiased, transcriptomic analysis, our work revealed that almost 30% of the ciliome was differentially expressed across analyzed embryonic tissues. Further examination of expression data suggested that ciliome heterogeneity correlated with tissue of origin (e.g. neural, facial and limb). In addition to spatial tissue-specific heterogeneity, we also identified dynamic expression of a subset of ciliary genes during osteogenic differentiation of multipotent neural crest cells. Thus, our work characterizes the ciliome as both tissue-specific and dynamic, and suggests that changes in the ciliome contribute to cell-type specific function. These findings support the possibility that heterogeneity of the ciliome not only contributes to tissue identity but could convey diverse cellular and molecular functions upon cells. We provide these data (Table S1) as a resource to the community on a searchable platform that can be curated and updated in real-time (https://research.cchmc.org/Ciliome_Gene_Expression/).

Cellular organelles (e.g. Golgi, mitochondria, ribosomes, etc.) are involved in several processes essential for cell survival and homeostasis, including energy production, protein production/packaging and response to external signals. Although organelles are traditionally thought to be ubiquitous and homogenous, and to play a conserved role across all tissues, this idea has recently been challenged with findings that some organelles, such as ribosomes, exhibit significant heterogeneity within different tissues. The compositions of core ribosomal proteins and ribosome associated proteins have been shown to vary significantly and to offer differential selectivity in translating specific pools of RNA transcripts that control different biological processes, e.g. cell metabolism and cellular differentiation, thereby adding an additional layer of genome-wide gene regulatory networks (Shi et al., 2017; Simsek et al., 2017).

Our study places primary cilia alongside ribosomes as heterogeneous organelles that contribute to tissue-specific identity. The implications of this finding are far-reaching. Current estimates based on phenotype and candidate gene studies suggest that as many as 400 undiagnosed ciliopathies exist (Schock and Brugmann, 2017). A comprehensive understanding of ciliary heterogeneity will allow us to better predict and diagnose previously uncharacterized ciliopathies. Second, understanding how various mutations to the ciliome impact the signaling environment will subsequently allow for more targeted approaches towards treating ciliopathies. Finally, modulating expression of heterogeneously expressed ciliary genes could serve as a targeted therapeutic option for ciliopathies with tissue-specific expression or any disease with aberrant cilia-dependent signaling.

In addition to increasing resources to treat ciliopathies, knowledge of the heterogeneous nature of the ciliome could be applied to several other disease classes. As primary cilia are important hubs for molecular signaling during development, the heterogeneity of the ciliome likely generates cilia with different sensitivities to molecular signals and mechano-sensory functions. Understanding the net impact of coordinated expression of ciliome subgroups could be used to engineer cells ‘tuned’ to receive specific molecular inputs. Generation of cells with acute sensitivities to molecular signals would be useful when using pharmacological compounds with off-target effects that have only proven effective at supraphysiological levels, or for guiding the differentiation of multipotent cells, such as cranial neural crest, into distinct lineages.

Our results suggest that tissue identity is a major molecular driver of ciliary heterogeneity. There is precedence for this suggestion. For example, mutations in *Mks1* affect cilia formation in a tissue and cell type-dependent manner (Weatherbee et al., 2009). Although cilia formation is impaired at the node, forelimb mesenchyme, lung mesenchyme and brains of *Mks1* mutants, cilia are still present on the multi-ciliated cells of the lung and bile ducts of the liver (Weatherbee et al., 2009). Additionally, although some *Mks1* mutant phenotypes (fusion and forking of the ribs, reduced mineralization in the skull and pulmonary hypoplasia) can easily be explained by a loss of Hh signaling, limb abnormalities reflect an expansion of Hh signaling, and the neural tube presents with phenotypes associated with both an expansion and reduction in Hh signaling (Weatherbee et al., 2009). This evidence further supports the hypothesis that not only are primary cilia heterogeneous across tissues, but that the function ciliary genes play is tissue dependent. As the loss of ciliary genes changes the relative abundance of Gli activator and repressor complexes, thereby changing the output of Hedgehog signaling (Huangfu and Anderson, 2005; Huangfu et al., 2003), loss of specific ciliary genes could elicit a tissue-specific response via their effect on processing Gli proteins.

Although the studies herein detail ciliome heterogeneity on a gene and protein expression level, additional levels of complexity must be considered to appreciate the full extent of heterogeneity among primary cilia. Post-translational modification (PTM) of ciliary proteins is another potential mechanism contributing to heterogeneity. It is well documented that axonemal tubulin is the most heterogenous tubulin due to a series of PTMs that include acetylation, detyrosination, glutamylation and glycosylation (Gaertig and Wloga, 2008). PTMs of tubulins contribute to variation within intraciliary trafficking, binding affinity and stability. Motor proteins, like kinesins and dyneins, can use tubulin PTMs as ‘road signs’ to direct the transport of cargoes to specific subcellular locations, including the axoneme (Verhey and Gaertig, 2007). Furthermore, functional studies have shown that loss of specific PTMs to either α or β-tubulin resulted in decreased binding of kinesin motor proteins to microtubules (Reed et al., 2006). Our analyses detected enrichment of genes related to tubulin glutamylation within neural tissues. These modifications have been related to microtubule stability (Bosch Grau et al., 2013) and more recently have been associated with the ability of neurons to release extracellular vesicles (Akella and Barr, 2021). Assessing whether specific modifications are over- or under-represented in a tissue-specific manner could build an additional layer of complexity on the concept of ciliome heterogeneity.

Perhaps the most intriguing finding of this study was the dynamic nature of the ciliome during cell differentiation. In profiling neural crest cells and their osteoblast derivatives, we found genes associated with skeletal pathologies as well as enrichment of genes that contribute to cytoskeletal and microtubule binding/organization. Interestingly, when examined in the functional context of the role of the cilium on neural crest versus skeletogenic cells, these expression changes suggest the transition of a primary cilium from an organelle that processes molecular signals into one that is mechanosensory in function (Nguyen and Jacobs, 2013; Yuan et al., 2015). Previous studies revealed a specialized cytoskeleton in *Drosophila* mechanosensory cells (Sun et al., 2021) and suggested that microtubule stability and connection to the cytoskeleton is a mechanism by which the cilium transmits external force (Hoey et al., 2012). Although the implications of these findings are important for the treatment and understanding of ciliopathies, it is unclear whether the changing expression of ciliary genes is driving cellular differentiation or whether it is a response to cellular differentiation. Alternatively, it is also conceivable that proteins within the ciliome serve additional non-ciliary roles that contribute to the observed phenotypes (Ferent et al., 2019). If driving differential expression of a subset of ciliary genes could dictate cell fate choices, several strategies could be envisioned for directing differentiation of multipotent cells such as neural crest. Our ongoing work focuses on this concept in a craniofacial context.

## MATERIALS AND METHODS

### Murine lines and husbandry

Mice used for bulk and scRNA-seq were maintained on a CD1 background or C57/BL6J (Jackson Laboratory stock no. 00664). Both male and female mice were used. A maximum of four adult mice were housed per cage, and breeding cages housed one male paired with up to two females. All mouse use was approved by the Institutional Animal Care and Use Committee (IACUC) and maintained by the Veterinary Services at Cincinnati Children's Hospital Medical Center. Noon on the day of finding a vaginal plug was considered as embryonic day 0.5.

### Cell culture, immunohistochemistry and quantification

All cell lines were maintained in a 37°C incubator with 5% CO_2_. NIH-3T3 cells were cultured in DMEM containing 10% FBS and 1×pen/strep mixture. NE-4C cells were purchased from ATCC, plated on poly-L-lysine-coated wells and cultured in EMEM containing the necessary supplements according to manufacturer's instructions (ATCC). Neural crest O9-1 cells (Millipore) were plated on Matrigel-coated wells and cultured in STO cell conditioned media containing necessary supplements according to the manufacturer's instructions (Millipore). Primary mouse embryonic fibroblasts (MEFs) were derived from E14.5 mouse embryos using standard methods.

Cells were fixed in 4% PFA at room temperature for 15 min and rehydrated in PBS containing 0.1% Triton X-100 (PBST). Blocking was performed for 1 h at room temperature using PBST containing 10% normal goat serum. Anti-Arl13b primary antibody (17711-1-AP, Proteintech) was used at a dilution of 1:1000 in 10% normal goat serum at 4°C overnight. Secondary antibody was used at a dilution of 1:1000 and staining was performed at room temperature for 1 h. The fixed and stained samples were mounted onto coverslips with mounting media.

All imaging was performed at the confocal imaging core at CCHMC. Images were obtained using an inverted Nikon A1R scanning confocal microscope equipped with a 488 nm and 405 nm laser. Cilia lengths, percentage and ciliogenesis rate were quantified using Nikon Elements analysis software. A minimum of 30 cilia were assayed in triplicate. To calculate the percentage of cells extending a cilium in 3T3, NE-4C and O9-1 cells, a minimum of 400 cells were counted in triplicate. Images were processed using ImageJ and one-way ANOVA statistics were analyzed in Prism 7.04 (Graphpad), in which *P*<0.05 was considered statistically significant.

### Cytochalasin D treatments

Cells were treated with cytochalasin D (Sigma C8273) at a concentration of 250 nM. At 0 or 2 h post-cytochalasin D exposure, cells were fixed in 4% PFA at room temperature for 15 min and rehydrated in PBS containing 0.1% Triton X-100 (PBST). Blocking was performed for 1 h at room temperature using PBST containing 10% normal goat serum. Anti Arl13b primary antibody (17711-1-AP, Proteintech) was used at a dilution of 1:1000 in 10% normal goat serum at 4°C overnight. Secondary antibody was used at a dilution of 1:1000 and staining was performed at room temperature for 1 h. The fixed and stained samples were mounted onto coverslips with mounting media. All imaging was performed at the confocal imaging core at CCHMC. The images were obtained using an inverted Nikon A1R scanning confocal microscope equipped with a 488 nm and a 405 nm laser. Images for publication were processed using ImageJ. Cilia lengths were quantified using Nikon Elements analysis software. The length of the cilia was measured from the base to the tip of primary cilia using the Arl13b marker signal in NIS Elements. To calculate the percentage of ciliated cells in the different cell lines, a minimum of 400 cells were counted in triplicate. Cilia lengths were measured for the different cell lines after 0 or 2 h of cytochalasin D treatment. A minimum of 30 cells were used to measure cilia length per time point in triplicate for each cell line. Rate of ciliary extension within the first 2 h of drug treatment was calculated from the difference in the mean ciliary lengths in cells treated with either 0 or 2 h of cytochalasin D from three independent experiments.

### Bulk RNA sequencing

Embryos were harvested and washed extensively with ice-cold DEPC-treated PBS. At E11.5, MXP (maxillary prominence), MNP (mandibular prominence), FNP (frontonasal prominence), limb, dB (dorsal brain) and vB (ventral brain) were dissected from the embryos and samples from multiple embryos were pooled for each tissue. Isolated tissue samples were homogenized, and total RNA was extracted using Trizol (Fisher Scientific) following the manufacturer's instructions. After quality control validation, samples were submitted for NGS sequencing. Six independent biological replicates were used for each tissue at E11.5. Paired-end sequencing was performed for each sample with a minimum of 20 million reads per library with a 75 bp read length. Library preparation and sequencing were performed at the DNA sequencing core at CCHMC for E11.5 samples.

For analysis, BAM files were loaded into Strand NGS. Mm10 build was used as the reference genome for the alignment. Samples passing Q30 quality were used for gene expression studies. Detection of novel genes and exons was allowed, and partial reads were also included. DESeq2 was used for normalization of expression data. Differential gene expression between any two E11.5 tissues was defined with a threshold of fold change of at least two and a 5% false discovery rate. Sequencing data have been deposited in GEO under accession number GSE147522.

### Bulk RNA-seq analysis

Raw reads from samples were analyzed using Computational Suite for Bioinformaticians and Biologists (CSBB-v2.0). Quality check, mapping and quantification of the reads was performed using FASTQC, Bowtie2 and RSEM, respectively, using CSBB wrapper. Reads were mapped to mouse transcriptome (mm10). Differentially expressed genes were obtained using R package (RUVSeq). Functional and pathway enrichment for DE genes were performed using ToppGene (Chen et al., 2009a,b, 2007).

### Single molecule fluorescent *in situ* hybridization (RNAScope)

Mouse embryos harvested at E11.5 were fixed with 4%PFA, embedded in paraffin wax and sectioned at 5 µm. Probes for *Pkp3*, *Rab3il1* and *Tulp3* were designed by ACDBio. Fluorescent *in situ* hybridization (FISH) was performed on the sections using RNAscope (ACDBio) following manufacturer's instructions. Samples were mounted using Prolong Gold anti-fade mounting medium (Life Technologies) and imaged on a Leica DFC310FX.

### Tissue dissection and western blot analysis

E11.5 mouse heads were incubated with 2 mg/ml Dispase II (ThermoFisher 17105041) in PBS on a rocker for 15 min at 37°C, as previously described (Li and Williams, 2013). Facial prominences were then dissected, and facial ectoderm was separated from underlying mesenchyme. Collected tissue was sonicated in cold RIPA buffer [50 mM Tris-HCl (pH 7.4), 1% NP-40, 0.25% sodium deoxycholate, 150 mM NaCl and 1 mM EDTA] containing protease and phosphatase inhibitors (ThermoFisher 78440). Protein extract was collected after 10 min full-speed centrifugation at 4°C. 30 µg of protein from each sample was used for western blot. Protein expression was visualized using Pkp3 (Novus Biologicals NBP1-97675, 1:100), Rab3il1 (Proteintech 17827-1-AP, 1:250) (Tong et al., 2021), Tulp3 (Proteintech 13637-1-AP, 1:500) (Hong and Hamilton, 2016), vinculin (Santa Cruz Biotechnology sc-73614, 1:2000), IRDye 800CW donkey anti-rabbit IgG (LICOR 926-32213, 1:2000) and IRDye 680RD donkey anti-mouse IgG (LICOR 925-68072, 1:2000) antibodies. Images were taken by LICOR Odyssey DLx.

### O9-1 culture and differentiation

O9-1 cells were cultured and differentiated as described previously (Ishii et al., 2012; Nguyen et al., 2018). O9-1 basal media, which consisted of DMEM containing 15% fetal bovine serum (FBS), 0.1 mM minimum essential medium (MEM) non-essential amino acids, 1 mM sodium pyruvate, 55 μM β-mercaptoethanol, 100 U/ml penicillin, 100 µg/ml streptomycin and 2 mM L-glutamine, was conditioned by STO feeder cells and filtered through 0.22 μm filter. Leukemia inhibitory factor (LIF, final concentration 1000 units/ml) and fibroblast growth factor-basic (bFGF, final concentration 25 ng/ml) were added immediately before use. Cells were passaged at confluency. To induce osteogenic differentiation, 200,000 O9-1 cells were seeded with O9-1 media in a 24-well plate. O9-1 media was replaced with osteogenic differentiation media [alpha-MEM supplemented with 0.1 µM dexamethasone, 100 ng/ml bone morphogenetic protein 2 (BMP2), 50 μg/ml ascorbic acid, 10 mM β-glycerophosphate, 10% FBS, 100 U/ml penicillin and 100 mg/ml streptomycin] when cells reached confluency. Media were changed daily for 9 days. Cells were harvested on day 10. Osteogenic differentiation was evaluated with Alizarin Red staining and qRT-PCR. Cells were authenticated via gene expression assays and were free of contamination.

### Alizarin Red staining

Cells were fixed with 4% PFA for 15 min at room temperature with gentle shaking. Cells were washed with PBS three times, stained with 2% Alizarin Red with gentle shaking for 12-16 h at 4°C, washed with water and imaged on a Leica M165FC microscope. For embryos, samples were fixed in 100% ethanol for 48 h, transferred to 100% acetone for 48 h and then stained with 0.005% Alizarin Red. Embryos were cleared with 1% KOH/20% glycerol and stored in 80% glycerol.

### OsteoImage mineralization assay

Cells were expanded for three passages before osteoblast differentiation. Briefly, cells were passaged at 5×10^3^ cells/cm^2^ on uncoated 24-well plates and cultured for 4-21 days in osteogenic medium [DMEM/F-12 with GlutaMAX (Gibco) supplemented with 10% FBS, 0.1 μM dexamethasone, 10 mM β-glycerophosphate and 200 μM ascorbic acid] with medium changes every other day. Control cells were cultured in medium without β-glycerophosphate and ascorbic acid. Cells were fixed with 4% PFA and analyzed for mineralization with the OsteoImage Mineralization Assay (Lonza) as per the manufacturer's directions. Briefly, after fixation cells were washed 1× wash buffer and stained with 1× staining reagent for 30 min at room temperature protected from light. Cells were washed with 1× wash buffer, stained with DAPI and imaged at 10× magnification with fluorescein filter set. Green fluorescent staining is proportional to the amount of mineralization present in the culture.

### Induced pluripotent stem cell (iPSC) culture and differentiation

iPSC colonies were maintained in mTesR medium (StemCell Technologies) and cultured on Matrigel (Corning, 354277). Cells were typically passaged once a week using GCDR (StemCell Technologies). iPSCs were differentiated into NCCs with the STEMDiff Neural Crest Differentiation Kit (StemCell Technologies) according to the company instructions. Briefly, one well of iPSCs was detached with Accutase (ThermoFisher) into single cells. Cells were resuspended in provided medium containing 10 µM Y-27632 (Cell Signaling, 13624S) and plated at 8.6×10^4^ cells/cm^2^ on Matrigel-coated 12-well plates. Cells were cultured with daily medium changes without Y-27632. On day 6, cells were passaged with Accutase into single cells (NCC P1) and maintained in Mesencult-ACF Plus medium (StemCell Technologies) supplemented with 2 mM L-glutamine until confluent. Cells were expanded for three passages before osteoblast differentiation. Briefly, cells were passaged at 5×10^3^ cells/cm^2^ on uncoated 24-well plates and cultured for 4-12 days in osteogenic medium [DMEM/F-12 with GlutaMAX (Gibco) supplemented with 10% FBS, 0.1 µM dexamethasone, 10 mM β-glycerophosphate and 200 µM ascorbic acid] with medium changes every other day. Cells were fixed with 4% PFA and stained with 2% Alizarin Red (pH 4.8).

### qRT-PCR

For O9-1 cells, RNA was isolated from cells using RNAqueous-Micro Total RNA Isolation Kit (Ambion, AM1931) following the manufacturer's protocol. 1 µg RNA was reverse transcribed into cDNAs using High-Capacity RNA-to-cDNA Kit (Applied Biosystems, 4387406) following the manufacturer's instructions. PowerUp SYBR Green Master Mix (Applied Biosystems, A25742) was used to run qRT-PCR. Serial dilutions (10 ng, 5 ng, 2.5 ng and 1.25 ng) of cDNA were used to test qRT-PCR efficiency. All qRT-PCR experiments were performed with at least three biological replicates, and each contained three technical replicates. Primers used for qRT-PCR are listed in Table S4.

For hiPSC-derived NCCs, RNA was extracted from pelleted cells and DNase treated (RNAqueous-Micro Total RNA kit, Invitrogen). cDNA was synthesized with the High-Capacity RNA-to-cDNA kit (Applied Biosystems). qRT-PCR was performed in triplicate using PowerUP SYBR Green Master Mix (Applied Biosystems, A25742) on Applied Biosystems QuantStudio 6 Flex Real-Time PCR System. All genes were normalized to Gapdh expression. NCCs at P3 were used for qPCR controls. Primers used for qRT-PCR are listed in Table S4.

### Generation and genotyping of CRISPR/Cas9 edited F0 embryos

Zygotes were collected from superovulated C57BL/6J females (The Jackson Laboratory, 000664) and Cas9 ribonucleoprotein (RNP) complexes were electroporated as previously described (Modzelewski et al., 2018). Cas9 RNPs were generated according to manufacturer recommendations (IDT). The following guide pairs were used in these experiments: Pcm1-u1, GGCCATGGATTTCAACTGTC; Pcm1-d1, GTCTGTACGGTATGATAGCC; Tmem107-u1, AGATCCGACCCATGGCCTCG; and Tmem107-d1, TCCAAACCTTATATGCGGGT. Guides were designed to generate deletions removing crucial region(s) of each gene. Founder embryos (F0) were collected at E14.5 and E17.5, and DNA was isolated from yolk sacs. Loss-of-allele (LOA) assays were designed using PrimerQuest Tool (IDT) to assess mutagenesis efficiency and Droplet Digital PCR (ddPCR, BioRad) was performed for copy number variant (CNV) analysis using ApoB as reference control. Primer-probe sets used for ddPCR assays are as follows: Pcm1-F, AGGCTGCTCTTCTAGCTTTG; Pcm1-probe, AGCATAAAGCAGAGCAAGCTATAGCTGT; Pcm1-R, GACTATCACATACCAGAGTCATCC; and Tmem107-F, TCACCCTGGGCCTCTTT; Tmem107-probe, TGGCTTCCTCTCAGGAGTCTCCA; Tmem107-R, AGGCTCTGGGTG-CTATTGA.

## Supplementary Material

10.1242/develop.201237_sup1 Supplementary information

## People behind the papers

This article has an associated People behind the papers interview with some of the authors.
