# Supplementary material for: Identification of a heterogeneous and dynamic ciliome during embryonic development and cell differentiation
Source: Development. 2023 Apr 27;150(8):dev201237. doi: 10.1242/dev.201237 (PMC10163354; doi:10.1242/dev.201237)
Supplement: Supplementary information [file develop-150-201237-s1.pdf]

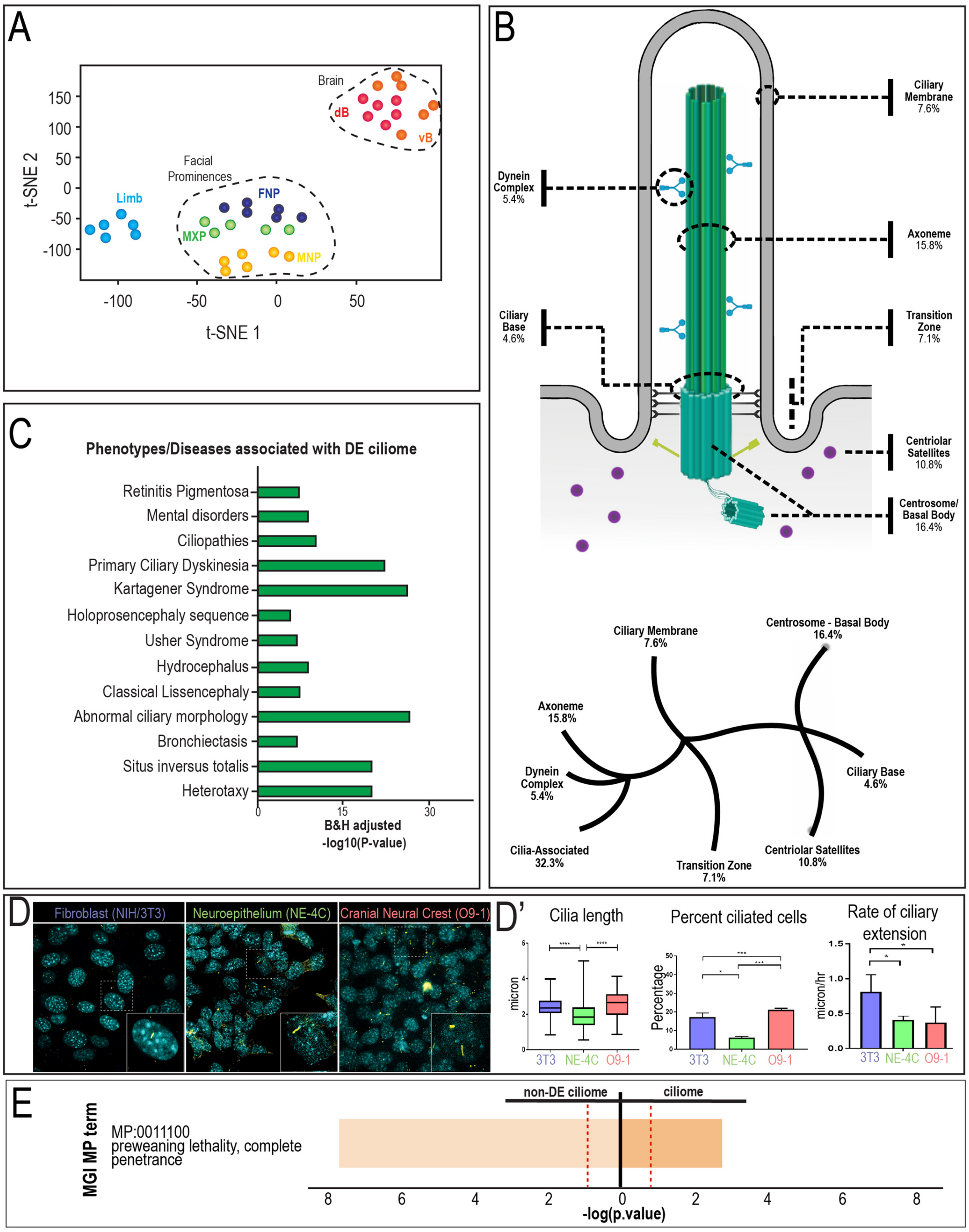

Fig. S1. (A) tSNE plot of sample clustering based upon bulk RNA-seq data. (B) Schematic of the ciliary apparatus and reactome showing the localization of proteins identified within the DE ciliome (if known). (C) GO terms associated with genes ofthe DE ciliome. (D) Immunohistochemistry for Arl13b in NIH3T3, NE4C and 09-1 cells. Dotted box indicates area of high magnification in inset. (D') Graphs showing the ciliary length, percentage of ciliated cells, and rate of ciliary extension quantification for NIH3T3, NE4C and 09-1 cells. (E) Enrichment analysis for MGI term "preweaning lethality, complete penetrance" between non-DE ciliome and full ciliome.

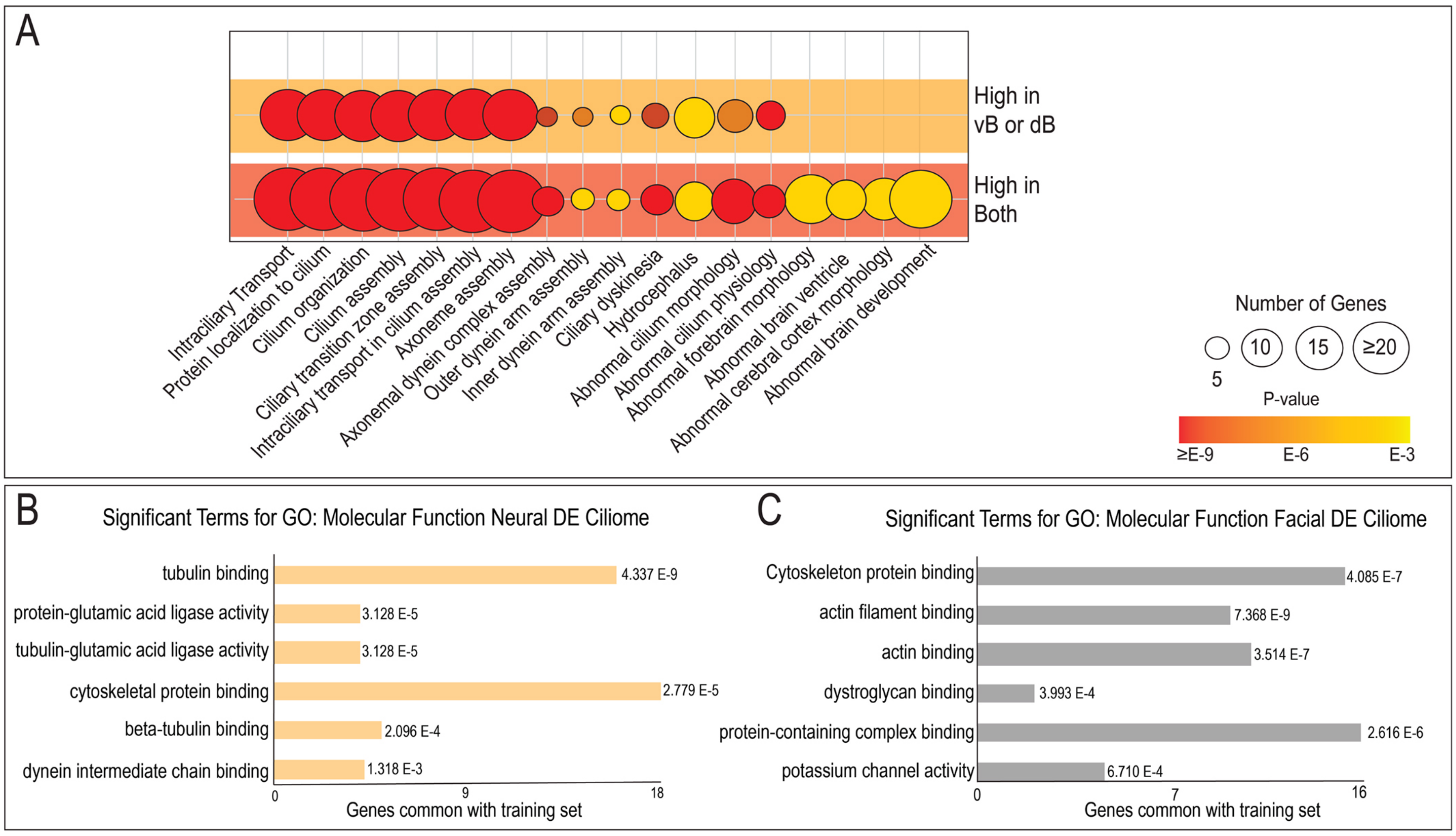

Fig. S2. DE ciliome in dorsal and ventral neural tissues. (A) Summary of GO terms enriched in ciliary genes robustly expressed in ventral (vB), dorsal (dB) or both neural tissues. Bubble size indicates number of ciliary genes per annotation, color reflects range of p-values. (B, C) GO-terms associated with molecular function of genes upregulated in neural and facial tissues (FDR < 0.05).

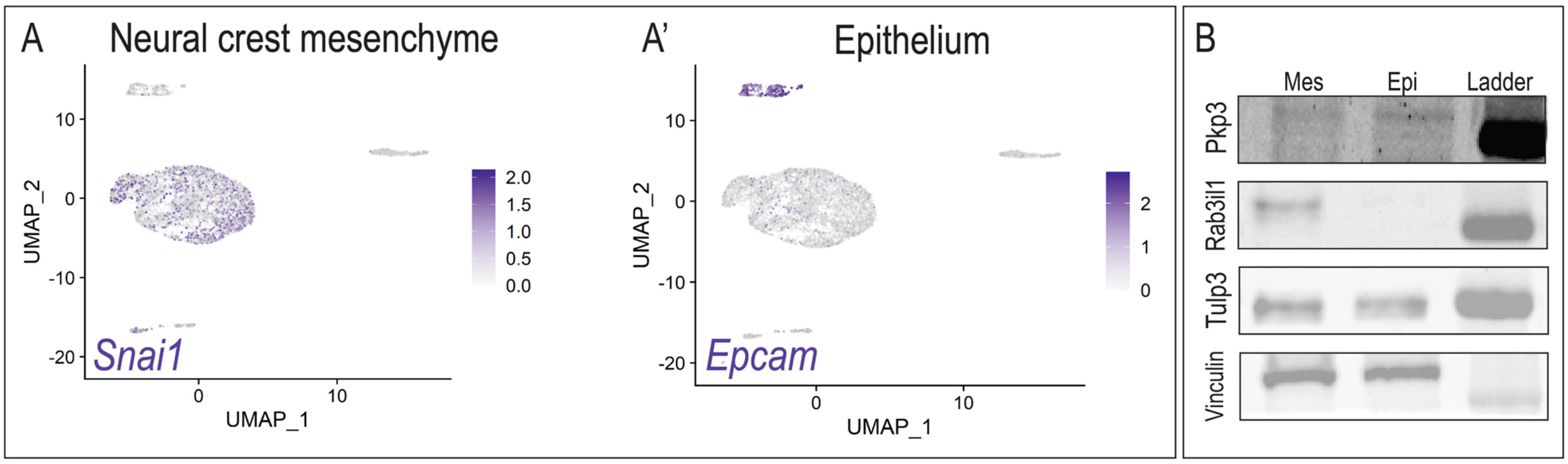

Fig. S3. (A) Feature plot of *Snai1* expression marking neural crest-derived mesenchyme in E11.5 MNP scRNA- seq. (A') Feature plot of *Epcam* expression marking epithelium in E11.5 MNP scRNA- seq. (B) Western blots for Pkp3, Rab3il1, and Tulp3 on micro-dissected and pooled E11.5 mesenchyme and epithelial tissue. Vinculin shown as loading control. Ladder in the rightmost co lumn.

Development • Supplementary information

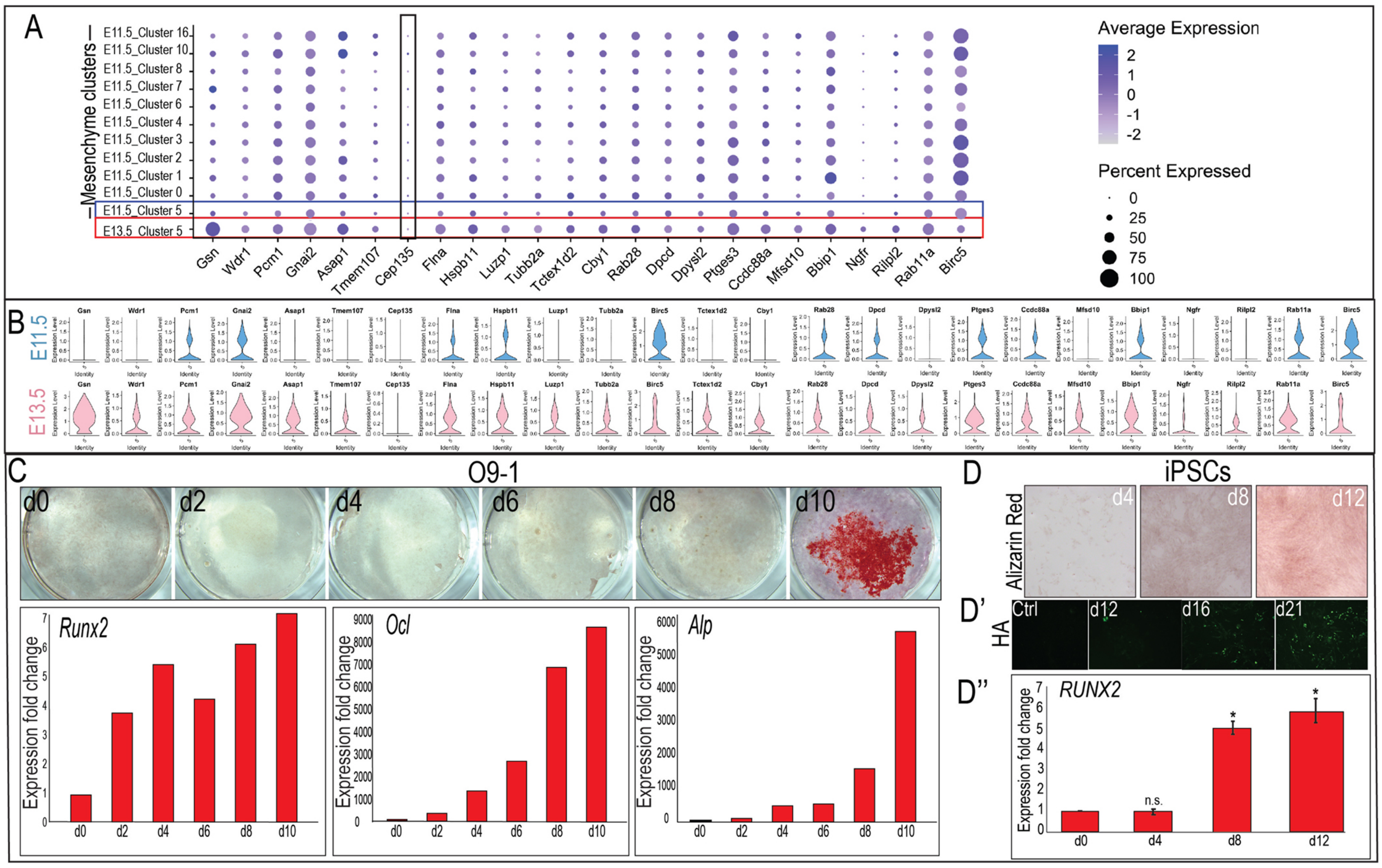

Fig. S4. (A) Dot plot depicting expression of genes with in the osteogenic ciliome in E11.5 mesenchymal clusters (Clusters 1-8, 10, 16) and E13.5 Cluster 5. Cluster 5 from E11.5 and Cluster 5 from E13.5 were compared as markers for skeletal progenitors were robustly expressed within these clusters. (B) Violin plots for genes of the osteogenic ciliome within E11.5 Cluster 5 and E13.5 Cluster 5. (C) Alizarin red staining and qPCR for osteoblast markers *Runx2,* Oc/, and *Alp* on 09-1 NCCs from day 0 to day 10 of osteogenic differentiation. (D) Alizarin red staining on iPSC-derived NCCs from day 4 to day 12 of osteogenic differentiation. (D") Hydroxyapatite expression via Osteoimage Mineralization Assay on control (without r1-glycerophosphate) and iPSC-derived NCCs from day 12 to day 21 of osteogenic differentiation. (D") Fold change expression of osteoblast markers *RUNX2* using qPCR from iPSC derived NCCs from dO to d12 of osteogenic differentiation.

Development • Supplementary information

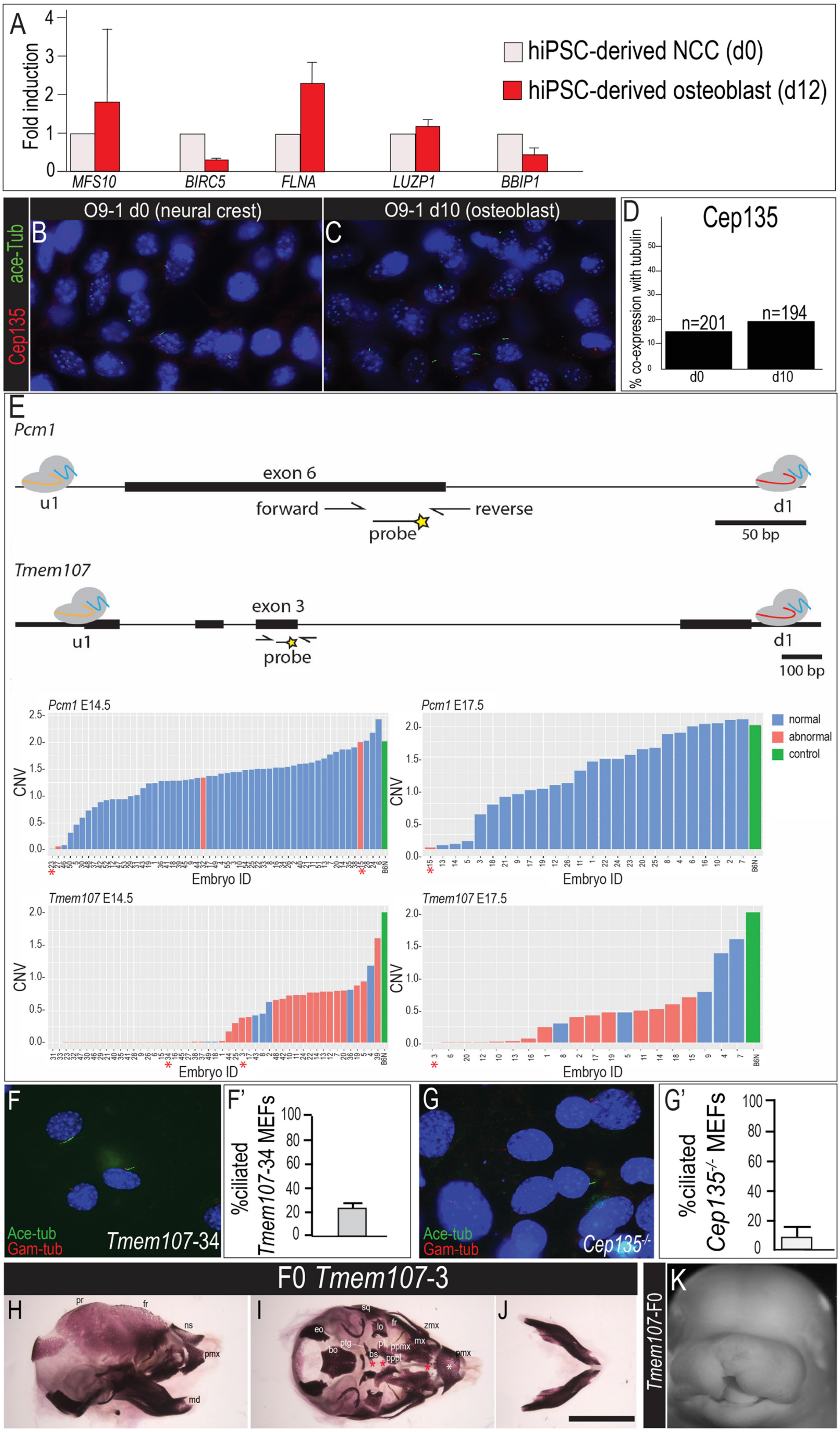

Development • Supplementary information

Fig. S5. Expression and knockout of genes within the osteogenic ciliome. (A) qPCR for select genes of the osteogenic ciliome during osteoblastic differentiation of hiPSC-derived NCCs (dO) and hiPSC-derived osteoblasts (d12). (8, C) lmmunostaining for Cep135 and acetylated-tubulin in 09-1 dO neural crest and 09-1 d10 osteoblast. (0) Quantification of cells co-expressing of Cep135 and acetylated-tubulin . (E) Schematic diagram of guide design and genotyping assay to determine copy number variation (CNV) using ddPCR for *Pcm1* and *Tmem107* knockout FO embryos. Bar plot shows individual CNV values for FO embryos with phenotype calls based upon gross morphological assessment at time of collection . Red asterisks denote embryos used for MEF analysis . (F) lmmunostaining for acetylated-tubulin and gamma-tubulin in MEFs derived from FO *Tmem107-34* embryos. (F') Quantification of percent ciliated cells in MEFs derived from FO *Tmem107-34* embryos. (G) lmmunostaining for acetylated-tubulin and gamma-tubulin in MEFs derived from *Cep135-1-* embryos. (G') Quantification of percent ciliated cells in MEFs derived from *Cep135-1-* embryos. (H-J) Lateral, ventral and mandibular views of Alizarin Red stained skulls from FO *Tmem107-3* embryos. Red asterisks indicate hypoplastic bone growth and clefting. White asterisk indicates fusion of premaxilla . (K) Frontal view of a FO *Tmem107* embryo with facial clefting. pr, parietal; ns, nasal; md, mandible; eo, exoccipital ; bo, basioccipital ; bs, basisphenoid ; ptg, pterygoid; pl, palatine; lo, lamina obturans ; sq, sqamosal ; fr, frontal ; zmx, zygomatic process of maxilla; mx, maxillary; ppmx, palatal process of maxilla; pppl, palatal process of palatine; pppx, palatal process of premaxilla ; pmx, premaxilla .

Development • Supplementary information

Table S1. Characterization of ciliome heterogeneity

Click here to download Table S1

Table S2. LOEUF decile (%) of DE and non-DE ciliome

LOEUF decile (%)	Number of ciliome genes	Number of DE ciliome genes	Number of non-DE ciliome genes	Percentage of DE ciliome genes	Percentage of non-DE ciliome genes	Ratio of Percentages (DE ciliome / non-DE ciliome)
0	137	30	107	11%	16%	0.68
10	103	27	76	10%	12%	0.86
20	83	24	59	9%	9%	0.98
30	109	31	78	11%	12%	0.96
40	134	41	93	15%	14%	1.06
50	109	33	76	12%	12%	1.05
60	98	34	63	13%	10%	1.30
70	76	24	52	9%	8%	1.11
80	58	19	39	7%	6%	1.17
90	22	9	13	3%	2%	1.67

Table S3. Characterization of the non-DE ciliome

Click here to download Table S3

Table S4. PCR primers

Gene	Forward	Reverse
Gapdh	5′-GGGTGTGAACCACGAGAAATA-3′	5′-GTCATGAGCCCTTCCACAAT-3′
Alp	5′-CTCCAAAAGCTCAACACCAATG-3′	5′-ATTTGTCCATCTCCAGCCG-3′
Ocl	5′-CACCTAGCAGACACCATGAG-3′	5′-GTTCACTACCTTATTGCCCTCC-3′
Runx2	5′-GCAATGCCTCCATTCAATCC-3′	5′-GGCAATATGTTGTCCTTGGAAC-3′
Bbip1	5′-CCTTCTGTGGACGTTTGGAGT-3′	5′-TGCTGGATGCAGTCTTTTGCG-3
Tubb2a	5′-CACCCCTTCTACAACCAGCA-3′	5′-GCTCATCGCTTATCACCTCCC-3′
Luzp1	5′-TTCCCCACTCCATGCTCAGG-3′	5′-CCAGTCACGATGGGCTGCTA-3′
Flna	5′-CCCTTTTCCCCGTACCGTGT-3′	5′-CTTTGCCTTTGCCTGCTGCT-3′
Rilpl2	5′-AGAGAAGGATGCTGTGGTTG-3′	5′-TGGTGATGGTTTTCAGTCTAGG-3′
Ccdc88a	5′-GACTGCCCGTGTCAGTCGAT-3′	5′-TGTTGCTCCCTAGACCTGCT-3′
Cby1	5′-GGATGCCTCTCTTTGGCAGC-3′	5′-GTTCCCGCGTTGACCGATCT-3′
Birc5	5′-AAGGAATTGGAAGGCTGGG-3′	5′-TTCTTGACAGTGAGGAAGGC-3′
Mfsd10	5′-GGGTAGACTGGTTTGCTTCAG -3′	5′-CCAGGTAATCAGAGGCAGC -3′
Asap1	5′-GAAGCAAATTTACCCAGCCC-3′	5′-TGATGTACTCTTTACGCACGG-3′
Pcm1	5′-AGTGATGTTTCTGCCGTCC-3′	5′-CTGAAGTGAGAAGCTGAGACG-3′
Tmem107	5′-GCTCTTGGCTCATCTGGTG-3′	5′-GGTTGTCCTGCTTTTCGTATTC-3′
Gsn	5′-AGGTCTTTGTCTGGGTTGG-3′	5′-TTTGCTGGATCTGTCTCGATG-3′
hBBIP1	5′ GCCTGAAGACAATCGGAGGG 3′	5′ CCACAAACAGTGGCCCTTGC 3′
hBIRC5	5′ CAAGGAGCTGGAAGGCTG 3′	5′ TTCTTGGCTCTTTCTCTGTCC 3′
hLUZP1	5′ CAGAAGGACCCAAAGTAGCC 3′	5′ CAGTTGCCTGTAGACACTGAG
hFLNA	5′ TGTGGCAATCAGAAGGTACG 3′	5′ ACATTCGATCTTAGCCTGCG 3′
hMFSD10	5′ TCTACTTCCTCTACCTCTTCCTG 3′	5′ GCATAGGCACCCTGGATG 3′

Development • Supplementary information
